# A likelihood ratio approach for identifying three-quarter siblings in genetic databases

**DOI:** 10.1038/s41437-020-00392-8

**Published:** 2021-01-15

**Authors:** Iván Galván-Femenía, Carles Barceló-Vidal, Lauro Sumoy, Victor Moreno, Rafael de Cid, Jan Graffelman

**Affiliations:** 1 Department of Computer Science, Applied Mathematics and Statistics, Universitat de Girona, Girona, Spain; 2 Genomes For Life - GCAT lab, Institute for Health Science Research Germans Trias i Pujol (IGTP), Can Ruti Campus, Badalona, Barcelona Spain; 3 High Content Genomics and Bioinformatics Unit, Institute for Health Science Research Germans Trias i Pujol (IGTP), Can Ruti Campus, Badalona, Barcelona Spain; 4 Oncology Data Analytics Program, Catalan Institute of Oncology (ICO), Badalona, Spain; 5 ONCOBELL Program, Bellvitge Biomedical Research Institute (IDIBELL), Barcelona, Spain; 6 Consortium for Biomedical Research in Epidemiology and Public Health (CIBERESP), Madrid, Spain; 7 Department of Clinical Sciences, University of Barcelona, Barcelona, Spain; 8 Department of Statistics and Operations Research, Universitat Politècnica de Catalunya, Barcelona, Spain; 9 Department of Biostatistics, University of Washington, Seattle, WA USA

**Keywords:** Genetic markers, Population genetics

## Abstract

The detection of family relationships in genetic databases is of interest in various scientific disciplines such as genetic epidemiology, population and conservation genetics, forensic science, and genealogical research. Nowadays, screening genetic databases for related individuals forms an important aspect of standard quality control procedures. Relatedness research is usually based on an allele sharing analysis of identity by state (IBS) or identity by descent (IBD) alleles. Existing IBS/IBD methods mainly aim to identify first-degree relationships (parent–offspring or full siblings) and second degree (half-siblings, avuncular, or grandparent–grandchild) pairs. Little attention has been paid to the detection of in-between first and second-degree relationships such as three-quarter siblings (3/4S) who share fewer alleles than first-degree relationships but more alleles than second-degree relationships. With the progressively increasing sample sizes used in genetic research, it becomes more likely that such relationships are present in the database under study. In this paper, we extend existing likelihood ratio (LR) methodology to accurately infer the existence of 3/4S, distinguishing them from full siblings and second-degree relatives. We use bootstrap confidence intervals to express uncertainty in the LRs. Our proposal accounts for linkage disequilibrium (LD) by using marker pruning, and we validate our methodology with a pedigree-based simulation study accounting for both LD and recombination. An empirical genome-wide array data set from the GCAT Genomes for Life cohort project is used to illustrate the method.

## Introduction

The detection of related individuals in genetic databases is of great interest in various areas of genetic research. Most obviously, it is of interest in forensic studies aiming at identifying relationships between individuals such as paternity tests (Evett and Weir, 1998) or sibling tests (Mo et al., 2016, Wang, 2004). Good high-resolution techniques for detecting related individuals are also of interest for genealogical research on family reconstruction (Staples et al., 2014). In conservation genetics, careful selection of unrelated individuals for breeding programs is needed (Oliehoek et al., 2006), requiring the estimation of pairwise genetic relationships. In genome-wide association studies (GWAS) that have become popular during the past two decades (Visscher et al., 2017), standard quality control filters are applied prior to genetic association analysis. The presence of cryptic relatedness violates the assumption of independent individuals in association modeling. For this reason, removing related individuals in the genetic database prior to the GWAS analysis is a common quality control step (Anderson et al., 2010).

Many methods for relatedness research are described in the literature. Most of them are based on the principle of allele sharing. Two individuals can share 0, 1, or 2 alleles for a diploid genetic marker. These alleles can be identical by state (IBS) or identical by descent (IBD). A scatterplot of the mean (${\bar{x}}_{IBS}$) and standard deviation (*s*_*IBS*_) of the number of IBS alleles over variants can be used to identify related pairs (Abecasis et al., 2001). Alternatively, a scatterplot of the proportions of sharing 0, 1, or 2 IBS alleles (*p*_0_, *p*_1_, *p*_2_) is also often used to detect related pairs (Rosenberg, 2006). In genetic studies, the probabilities of sharing 0, 1, and 2 IBD alleles (*k*_0_, *k*_1_, *k*_2_) can be estimated and used for relationship inference, since their theoretically expected values are known for the standard relationships (see Table 1). For example, parent–offspring pairs have (*k*_0_, *k*_1_, *k*_2_) = (0, 1, 0) and full siblings have (*k*_0_, *k*_1_, *k*_2_) = (0.25, 0.50, 0.25). For a given pair of individuals, these probabilities can be estimated by maximum likelihood (Milligan, 2003, Thompson, 1975, 1991), by the method of moments (Purcell et al., 2007) or with robust estimators (Manichaikul et al., 2010). From these probabilities, the kinship coefficient, defined as *ϕ* = *k*_1_/4 + *k*_2_/2, can be obtained. The kinship coefficient can be used to remove individuals with first degree (parent–offspring (PO) or full siblings (FS)) and second-degree relationships (half-siblings, avuncular or grandparent–grandchild) by retaining only pairs with *ϕ* < 1/16. In addition, third-degree relationships (first cousins (FC)) can be eliminated by retaining only pairs with *ϕ* < 1/32 (Anderson et al., 2010). All these methods have in common that the inference of the family relationships is based on the judgment of the analyst of the point estimates (${\hat{k}}_{0},{\hat{k}}_{1},{\hat{k}}_{2},\hat{\phi }$) or of a graphical representation ((${\bar{x}}_{IBS}$,*s*_*IBS*_), (*p*_0_, *p*_1_, *p*_2_) or (${\hat{k}}_{0},{\hat{k}}_{1},{\hat{k}}_{2}$)) (Galvan-Femenia et al., 2017).

**Table 1:** Degree of relationship (R), kinship coefficient (*ϕ*), and probability of sharing zero, one or two alleles identical by descent (*k*_0_, *k*_1_, *k*_2_).

			Probability of IBD sharing		
Type of relative	R	*ϕ*	*k*_0_	*k*_1_	*k*_2_
Monozygotic twins (MZ)	0	1/2	0	0	1
Parent–offspring (PO)	1	1/4	0	1	0
Full siblings (FS)	1	1/4	1/4	1/2	1/4
Three-quarter siblings (3/4S)	–	3/16	3/8	1/2	1/8
Half-siblings/grandchild–grandparent/niece/nephew–uncle/aunt (2nd)	2	1/8	1/2	1/2	0
First cousins (FC)	3	1/16	3/4	1/4	0
Unrelated (UN)	*∞*	0	1	0	0

The sample size used in genetic studies, GWAS in particular, is progressively increasing owing to large human sequencing projects that involve genetic data from hundreds of thousands of individuals such as UK Biobank (Bycroft et al., 2018), gnomAD (Karczewski et al., 2020), TOPMed (Taliun et al., 2019), and DiscovEHR (Staples et al., 2018) among others. With such large databases, it becomes increasingly likely that in-between 1st and 2nd degree, and in-between 2nd and 3rd-degree relationships are found. Such in-between relationships are mostly ignored in a relatedness analysis, which typically mostly focus on 1st, 2nd, and 3rd-degree relationships. In this paper, we therefore develop a likelihood ratio (LR) approach that will allow us to identify three-quarter siblings (3/4S), a family relationship whose individuals share fewer alleles than 1st-degree relationships but more alleles than 2nd-degree relatives (Table 1). A 3/4S pair has one common parent, whereas their unshared parents have a first-degree relationship (FS or PO; see Graffelman et al. 2019 Fig. S2). The IBD probabilities for 3/4S are (*k*_0_, *k*_1_, *k*_2_) = (3/8, 1/2, 1/8) and their kinship coefficient is *ϕ* = 3/16. A detailed derivation of these probabilities is shown in Appendix A. A 3/4S relationship is not very common, but is more likely to be present in GWAS studies with ever-increasing sample sizes. The 3/4S relationship has received very little attention in the literature, and the aim of this paper is to develop tools that distinguish 3/4S from full siblings and second-degree relatives.

The remainder of this paper is structured as follows. Section “Methods and materials” develops a LR approach for identifying three-quarter siblings. Section “Simulations” evaluates the LR approach in a simulation study. Section “Case study” illustrates our approach with genome-wide SNP array data from the GCAT Genomes for Life project cohort. Finally, we end the article with a discussion of the proposed methodology.

## Methods and materials

### Overview of the likelihood of a relationship

A detailed derivation of the likelihood of having a given relationship is given by Wagner et al. (2006). In brief, let *n* be the number of individuals in a non-inbred homogeneous population and assuming absence of population structure. We consider biallelic genetic variants with alleles *A* and *B* having allele frequencies *p* and *q*, respectively. Let *G*_1_/*G*_2_ be the genotypes for a pair of individuals, let *k*_*m*_ with *m* = 0, 1, 2 be their IBD probabilities (shown in Table 1) and let *R* be their family relationship. Then, the probability of observing *G*_1_/*G*_2_, given *R* is:

1 $$\begin{array}{lll}P({G}_{1}/{G}_{2}| R)\,=\,P({G}_{1}/{G}_{2}| m=0){k}_{0}\\ \qquad\qquad\qquad\quad+\,P({G}_{1}/{G}_{2}| m=1){k}_{1}\\ \qquad\qquad\qquad\quad+\,P({G}_{1}/{G}_{2}| m=2){k}_{2}.\end{array}$$

The terms *P*(*G*_1_/*G*_2_∣*m* = 0), *P*(*G*_1_/*G*_2_∣*m* = 1) and *P*(*G*_1_/*G*_2_∣*m* = 2) are the probabilities of observing each pair of genotypes given the number of IBD alleles (Table 2).

**Table 2:** Possible pairs of biallelic genotypes and the probability of each pair given the number of alleles identical by descent (*m*).

*G*_1_/*G*_2_	*m* = 0	*m* = 1	*m* = 2
*AA*/*AA*	*p*^4^	*p*^3^	*p*^2^
*AA*/*AB*	2*p*^3^*q*	*p*^2^*q*	0
*AA*/*BB*	*p*^2^*q*^2^	0	0
*AB*/*AB*	4*p*^2^*q*^2^	*pq*	2*pq*

We assume that the order of the genotypes is irrelevant, i.e., the probabilities for *G*_1_*/G*_2_ and *G*_2_*/G*_1_ are the same.

### The LR approach for identifying three-quarter siblings

The LR approach has been widely used for relatedness research during the last decades (Boehnke and Cox, 1997, Heinrich et al., 2016, Katki et al., 2010, Kling and Tillmar, 2019, Thompson, 1986, Weir et al., 2006). In brief, the LR approach is based on the contrast of two hypotheses, one in the numerator, *H*_*i*_; and the other one in the denominator, *H*_*j*_. The larger the LR, the more plausible is *H*_*i*_; whereas the smaller the LR, the more plausible is *H*_*j*_. For relatedness research, we consider the ratio of the probabilities from Eq. 1 according to the hypothesis of the *R*_*i*_ and *R*_*j*_ relationships. That is:

2 $$LR({R}_{i},{R}_{j}| {G}_{1}/{G}_{2})=\frac{P({G}_{1}/{G}_{2}| {R}_{i})}{P({G}_{1}/{G}_{2}| {R}_{j})}$$

Here we consider the FS, 3/4S, 2nd, and unrelated (UN) relationships and calculate three LR having FS, 3/4S, or 2nd in the numerator and having the UN relationship in the denominator. The common denominator makes the LR values comparable in order to distinguish 3/4S from FS and 2nd degree. The inference of relatedness for each pair of individuals is based on the largest LR value in the FS ~ UN, 3/4S ~ UN, and 2nd ~ UN ratios. The LRs are shown in Table 3, depending on the observed genotypes of a pair of individuals. Most of these ratios are derived in Heinrich et al. (2016), and the new results for 3/4S are shown in Appendix B. The *e* parameter from the PO ~ UN ratio in Table 3 is a small number (i.e., 0.001) used to account for genotype errors and de novo mutations if the genotype combination does not occur. In this way, the LR cannot be zero. For *S* biallelic SNPs, the LR can be obtained by multiplying the *LR*_*s*_ across independent markers and by dividing by the number of SNPs. It is convenient to work in a logarithmic scale such that:

3 $$\begin{array}{lll}{\mathrm{log}\,}_{10}(LR)\,=\, \displaystyle \frac{1}{S}{\mathrm{log}\,}_{10}\left(\mathop{\prod }\limits_{s=1}^{S}L{R}_{s}({R}_{i},{R}_{j}| {G}_{1}/{G}_{2})\right)\\ \qquad\qquad\quad= \displaystyle \frac{1}{S}\mathop{\sum }\limits_{s=1}^{S}{\mathrm{log}\,}_{10}\left(L{R}_{s}({R}_{i},{R}_{j}| {G}_{1}/{G}_{2})\right),\end{array}$$

which corresponds to the logarithm of the geometric mean of the LRs. Obtained LRs are subject to uncertainty. To assess this uncertainty, we propose to apply bootstrap resampling (Efron and Tibshirani, 1994). This allows the construction of 95% bootstrap confidence intervals for the LRs, which are helpful to assess which relationship is the most likely one for a given pair.

**Table 3:** Likelihood ratio (LR) for relatedness research for biallelic SNPs.

LR	*AA*/*AA*	*AA*/*AB*	*AB*/*AB*	*AA*/*BB*
PO ~ UN	$\frac{1}{p}$	$\frac{1}{2p}$	$\frac{1}{4pq}$	$\frac{e}{{p}^{2}{q}^{2}}$
FS ~ UN	$\frac{1}{4}+\frac{1}{2p}+\frac{1}{{(2p)}^{2}}$	$\frac{1}{4}+\frac{1}{4p}$	$\frac{1}{4}+\frac{1}{4pq}$	$\frac{1}{4}$
3/4S ~ UN	$\frac{3}{8}+\frac{1}{2p}+\frac{1}{8{p}^{2}}$	$\frac{3}{8}+\frac{1}{4p}$	$\frac{3}{8}+\frac{3}{16pq}$	$\frac{3}{8}$
2nd ~ UN	$\frac{1}{2}+\frac{1}{2p}$	$\frac{1}{2}+\frac{1}{4p}$	$\frac{1}{2}+\frac{1}{8pq}$	$\frac{1}{2}$
FC ~ UN	$\frac{3}{4}+\frac{1}{2p}$	$\frac{3}{4}+\frac{1}{4p}$	$\frac{3}{4}+\frac{1}{16pq}$	$\frac{3}{4}$

The considered LR are PO, FS, 3/4S, 2nd, or FC relationships in the numerator and the UN relationship in the denominator. The LR values depend on the observed genotypes of a pair of individuals and the allele frequencies *p* and *q* of the population under study. The *e* parameter is used to account for genotype errors and de novo mutations if the genotype combination does not occur (Heinrich et al., 2016). We assume that the order of the genotypes is irrelevant, i.e., the LR for *G*_1_/*G*_2_ and *G*_2_/*G*_1_ is the same.

### Materials

We test our method for detecting 3/4S with data from the GCAT Genomes for Life cohort project (Obón-Santacana et al., 2018). In brief, the GCAT project is a prospective study that includes ~20K participants recruited from the general population of Catalonia, a Western Mediterranean region in the Northeast of Spain. A subset of 5459 participants was genotyped using the Infinium Expanded Multi-Ethnic Genotyping Array (MEGAEx) (ILLUMINA, San Diego, California, USA). In the present work, we consider 5075 GCAT participants of Caucasian ancestry and 756,003 SNPs that passed strict quality control (Galvan-Femenia et al., 2018). A previous relatedness research analysis of this dataset reported 63 FS, eight 3/4S, and 12 2nd-degree candidate pairs (Graffelman et al., 2019).

## Simulations

In this section, we evaluate the likelihood ratio approach to distinguish 3/4S from FS and 2nd relationships by using simulated data. Pedigrees were simulated from the genetic data of the individuals of the GCAT project, using the ped-sim method of Caballero et al. (2019). We apply this method in order to account for recombination by using sex-specific genetic maps (Bherer et al., 2017) and also a crossover interference model (Campbell et al., 2015). The simulations were carried out as follows. First, we identified 4147 potentially unrelated individuals with kinship coefficient <0.025. From these individuals, we retained 537,488 autosomal SNPs with minor allele frequency (MAF) > 0.01, Hardy–Weinberg exact mid *p* value > 0.05 (Graffelman and Moreno, 2013) and missing call rate zero. Genotypes of the unrelated individuals were phased with SHAPEIT4 (Delaneau et al., 2019) and were used as input for the ped-sim method. Then, we simulated 500 pedigrees containing one FS pair and 500 pedigrees containing one 3/4S pair. In total, we used 3000 random GCAT individuals as founders to generate 3000 artificial individuals. The number of simulated related pairs were 4,000 PO, 500 FS, 500 3/4S and 3,500 2nd degree from a total of 17,997,000 of pairs. To estimate the IBD probabilities and the kinship coefficient for these simulated pairs we used 27,087 SNPs obtained by retaining variants with MAF > 0.40 and by LD pruning, requiring markers to have low pairwise correlation (*r*^2^ < 0.20).

**Fig. 1:**
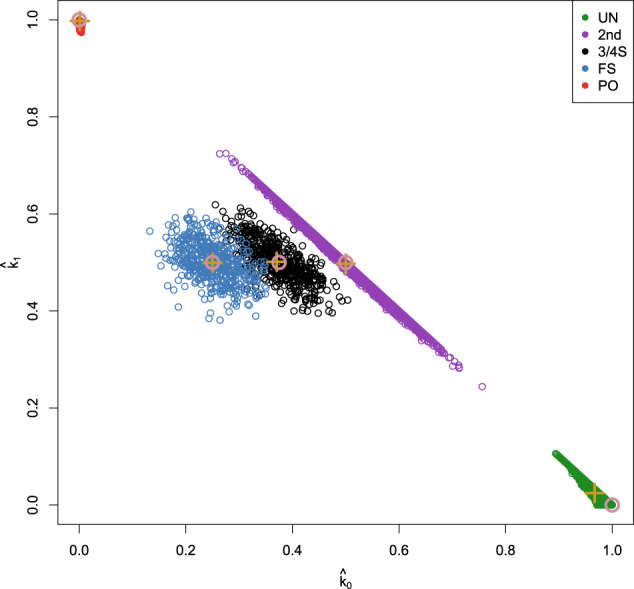
$({\hat{k}}_{0},{\hat{k}}_{1})$-plot of ~18 million pairs of simulated individuals using 27,087 SNPs. UN: unrelated; 2nd: second-degree relationships; 3/4S: three-quarter siblings. FS: full siblings; PO: parent–offspring. Brown open dots represent theoretical IBD probabilities; brown + signs the average of the corresponding group.

Figure 1 shows the $({\hat{k}}_{0},{\hat{k}}_{1})$-plot for these simulated pairs of individuals. The IBD probabilities were estimated with the PLINK software (Purcell et al., 2007). As expected, the estimated IBD probabilities are close to the expected theoretical values from Table 1 for most pairs of individuals. In Fig. 1, the 3/4S relationships show good separation from 2nd-degree relationships but mix to some extent with FS pairs. Estimated IBD probabilities appear to be centered on their expected values for FS, 3/4S, and 2nd-degree pairs, and have larger variance then PO and UN pairs. The discriminative power of our method crucially depends on the variance of these estimated probabilities (Hill and Weir, 2011).

**Fig. 2:**
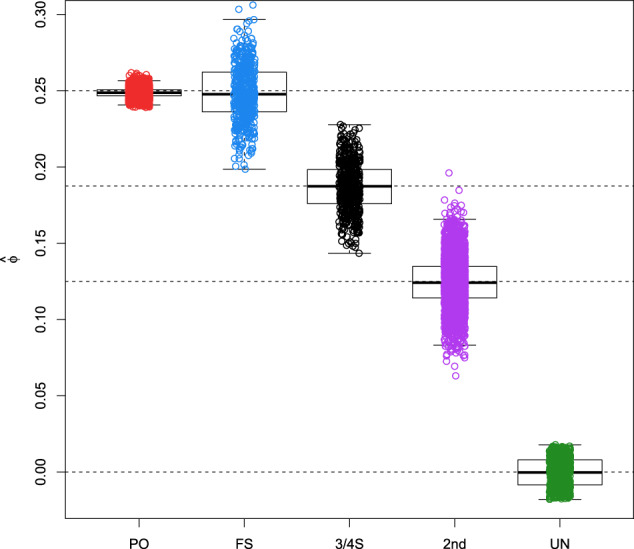
Boxplot of kinship estimates of ~18 million pairs of simulated individuals using 27,087 SNPs.

**Fig. 3:**
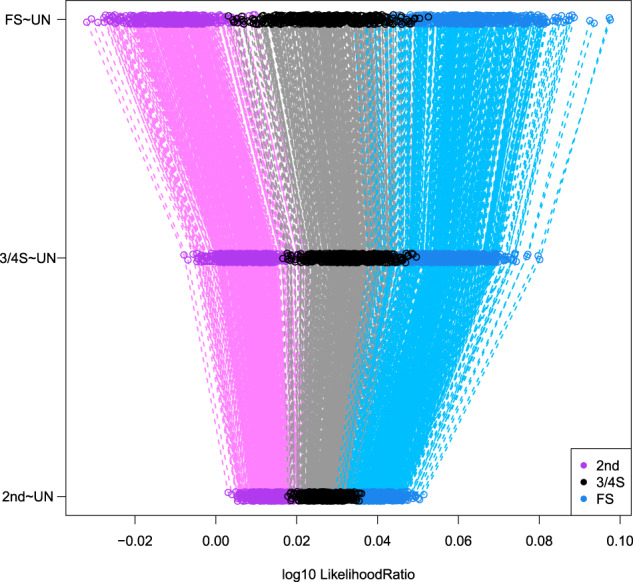
Log10 likelihood ratio approach of the simulated 2nd, 3/4S, and FS pairs (500 for each relationship) using 27,087 SNPs. Note the larger than sign shaped (“ > ”) pattern (gray dashed lines) for most 3/4S pairs.

Boxplots of the kinship estimator recently proposed by Goudet & Weir (Goudet et al. (2018), Weir and Goudet (2017)) shown in Fig. 2 clearly show a difference in median for 3/4S and 1st- and 2nd-degree relationships, though the distribution of the kinship coefficient of the 3/4S overlaps with those of 1st and 2nd-degree pairs. Also, kinship coefficients can be identical for different relationships, as is the case for PO and FS. Therefore, according to Eq. (3), we calculate the FS ~ UN, 3/4S ~ UN, and 2nd ~ UN likelihood ratios for 500 2nd, 500 3/4S, and 500 FS simulated pairs. Figure 3 shows that FS pairs mostly have the largest LR values in the FS ~ UN ratio, 3/4S pairs mostly have the largest LR values in the 3/4S ~ UN ratio and 2nd-degree pairs mostly have largest LR in the 2nd ~ UN. Note the plotted data profile shaped in a “greater-than” sign (“>”) pattern suggesting the inference of 3/4S for most 3/4S pairs. In fact, the correct classification rate of the LR approach for the 2nd, 3/4S and FS simulated pairs is 500/500 = 1, 479/500 = 0.958 and 475/500 = 0.95, respectively. When comparing the correct classification rate of the LR approach with the LR-kinbiplot approach (Graffelman et al., 2019) based on 500 FS, 500 3/4S, 3,500 2nd, and 5,000 UN simulated pairs (Fig. S1), we observe slightly lower classification rates for 3/4S (478/500 = 0.956) and FS (468/500 = 0.936) using linear discriminant analysis and slightly better classification rates for 3/4S (481/500 = 0.962) and FS (483/500 = 0.966) when using quadratic discriminant analysis as a predictive model. These simulations show the proposed LR approach to be useful for distinguishing 3/4S relationships from FS and 2nd-degree relationships, and to have similar performance to the previously proposed LR-kinbiplot approach.

## Case study

**Fig. 4:**
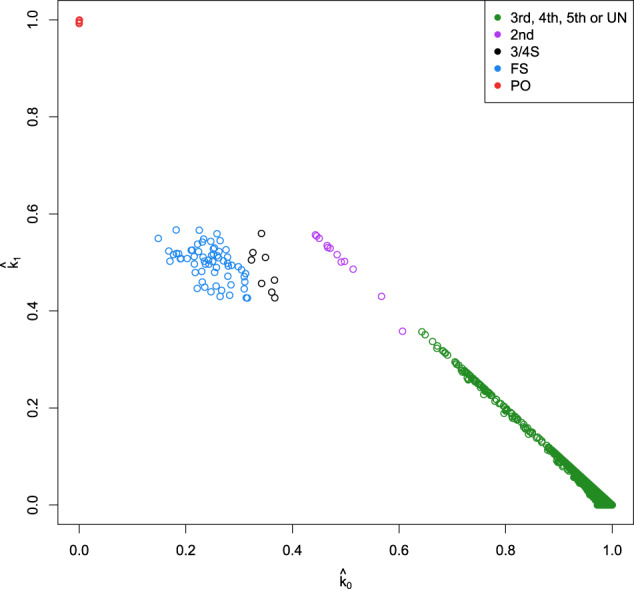
$({\hat{k}}_{0},{\hat{k}}_{1})$-plot of the GCAT cohort for 5075 individuals and 26,006 SNPs (MAF > 0.40, LD-pruned, HWE exact mid *p* value > 0.05, and missing call rate 0). 3rd, 4th, 5th, or UN: third, fourth, fifth-degree relationships or unrelated; 2nd: second-degree relationships; 3/4S: three-quarter siblings; FS: full siblings; PO: parent–offspring.

**Fig. 5:**
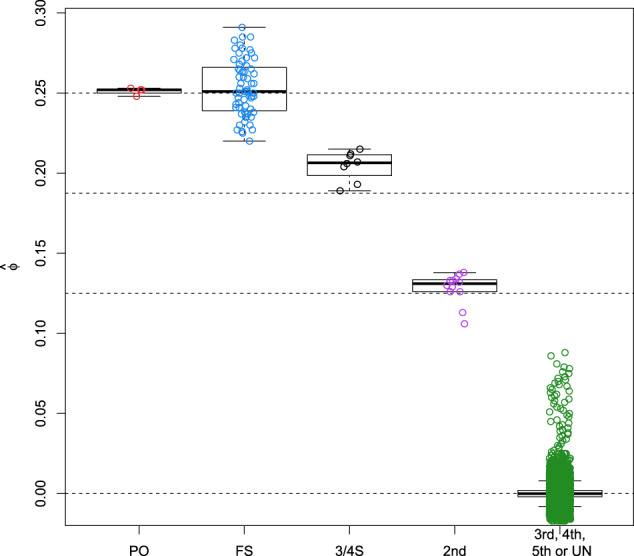
Boxplot of kinship estimates of the GCAT cohort for 5,075 individuals and 26,006 SNPs (MAF > 0.40, LD-pruned, HWE exact mid *p* value > 0.05, and missing call rate 0).

In this section, we apply the likelihood ratio approach to genome-wide SNP array data from the aforementioned GCAT project. Graffelman et al. (2019, Table 5 and Fig. 7) suggested this database to contain eight 3/4S pairs using a log-ratio biplot approach combined with discriminant analysis (LR-kinbiplot). Figures 4 and 5 show the $({\hat{k}}_{0},{\hat{k}}_{1})$-plot and boxplots of kinship estimates of the GCAT data. The IBD probabilities were estimated with the PLINK software, whereas the kinship coefficient was estimated by the estimator proposed by Weir and Goudet (2017). The colors for the FS, 3/4S, and 2nd-degree pairs in both Figures were assigned according to the relationship inferred by the LR approach. Figure 4 shows, like the simulations, a larger variance for FS pairs, and smaller variances for PO and UN pairs.

**Fig. 6:**
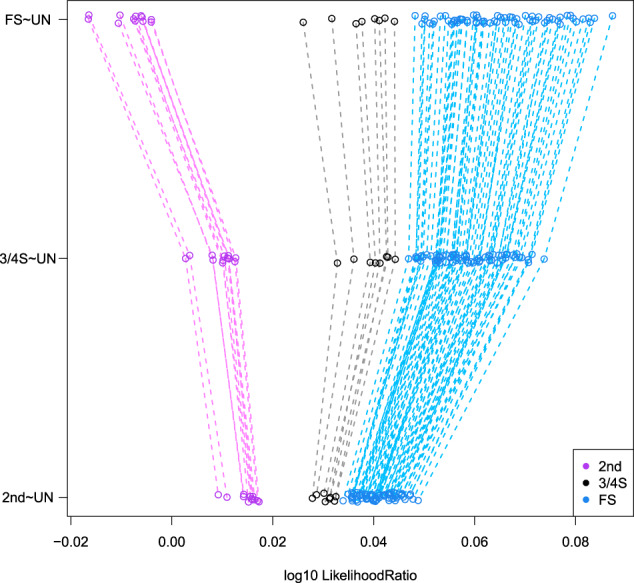
Log10 likelihood ratio approach of the presumably 2nd, 3/4S, and FS pairs from the GCAT cohort using 26,006 SNPs (MAF > 0.40, LD-pruned, HWE exact mid *p* value > 0.05, and missing call rate 0).

**Fig. 7:**
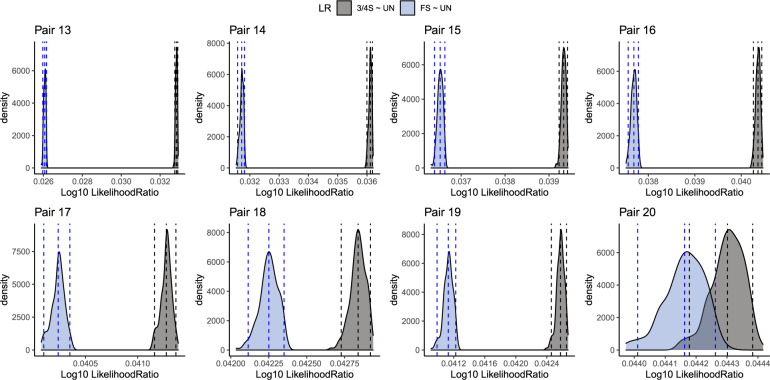
Bootstrap distribution of the LR for eight presumably 3/4S pairs of the GCAT project. Vertical dashed lines indicate the average LR values and the 95% bootstrap confidence interval limits.

Figure 6 shows the LR ratio values for the three relationships (FS ~ UN, 3/4S ~ UN and 2nd ~ UN ratios) on the horizontal axis, for the presumably FS, 3/4S and 2nd pairs from the GCAT project. The LR analysis reveals eight 3/4S pairs (black color) that have the ‘greater-than’ sign (“>”) shaped pattern, because the largest LR values are obtained for the 3/4S ~ UN ratio. All inferred FS pairs (blue color) have a monotonously increasing shaped pattern (“/”) since the largest LR values are obtained for the FS ~ UN ratio; and all 2nd-degree pairs have a monotonously decreasing pattern (“\”) since the largest LR values are obtained for the 2nd ~ UN ratio. Table 4 shows the LR values for each pair which confirm that there are eight 3/4S pairs in concordance with the LR-kinbiplot approach. We used bootrapping to assess the amount of uncertainty in the LRs. The bootstrap distribution of the LR for the eight hypothesized 3/4S pairs is shown in Fig. 7. This plot shows seven pairs having the entire bootstrap distributions for the two relationships completely separated, and these pairs therefore clearly do not correspond to FS pairs. For one pair (20) the 3/4S relationship is most likely, for having on average the largest LR; however, given the overlap of the two distributions, the evidence for a 3/4S relationship is less compelling for this pair.

**Table 4:** Likelihood ratio inference (LR approach) for the presumably 2nd, 3/4S, and FS pairs from the GCAT cohort.

Pair	IID	Sex	IID	Sex	${\hat{k}}_{0}$	${\hat{k}}_{1}$	${\hat{k}}_{2}$	$\hat{\phi }$	LR-kinbiplot	FS~UN	3/4S~UN	2nd~UN	LR approach
1	REL_00178	F	REL_01132	F	0.61	0.36	0.04	0.107	2nd	−0.0165	0.0027	**0.0092**	2nd
2	REL_02227	F	REL_00865	M	0.57	0.43	0.00	0.109	2nd	−0.0164	0.0035	**0.0109**	2nd
3	REL_04137	F	REL_03163	M	0.51	0.49	0.00	0.122	2nd	−0.0103	0.0082	**0.0142**	2nd
4	REL_04126	F	REL_02089	F	0.50	0.50	0.00	0.126	2nd	−0.0106	0.0080	**0.0143**	2nd
5	REL_04141	F	REL_02030	M	0.49	0.50	0.01	0.129	2nd	−0.0072	0.0101	**0.0152**	2nd
6	REL_02092	M	REL_00587	F	0.48	0.52	0.00	0.129	2nd	−0.0073	0.0104	**0.0158**	2nd
7	REL_02212	M	REL_04828	F	0.47	0.53	0.00	0.132	2nd	−0.0061	0.0111	**0.0161**	2nd
8	REL_00603	F	REL_00189	F	0.47	0.53	0.00	0.134	2nd	−0.0076	0.0101	**0.0156**	2nd
9	REL_03666	M	REL_02902	M	0.47	0.53	0.00	0.134	2nd	−0.0057	0.0112	**0.0160**	2nd
10	REL_00132	F	REL_00707	M	0.45	0.55	0.00	0.137	2nd	−0.0059	0.0113	**0.0164**	2nd
11	REL_02058	F	REL_03610	F	0.45	0.55	0.00	0.139	2nd	−0.0041	0.0125	**0.0170**	2nd
12	REL_01692	F	REL_00010	F	0.44	0.56	0.00	0.139	2nd	−0.0041	0.0127	**0.0173**	2nd
13	REL_03969	M	REL_00271	M	0.34	0.56	0.10	0.189	3/4S	0.0260	**0.0328**	0.0279	3/4S
14	REL_03803	F	REL_02343	M	0.35	0.51	0.14	0.198	3/4S	0.0317	**0.0361**	0.0287	3/4S
15	REL_03924	M	REL_03023	F	0.37	0.46	0.17	0.201	3/4S	0.0365	**0.0393**	0.0301	3/4S
16	REL_00083	M	REL_02333	M	0.33	0.52	0.15	0.207	3/4S	0.0377	**0.0403**	0.0313	3/4S
17	REL_01344	M	REL_02408	F	0.36	0.44	0.20	0.210	3/4S	0.0402	**0.0412**	0.0304	3/4S
18	REL_04189	M	REL_00775	M	0.36	0.44	0.20	0.210	3/4S	0.0422	**0.0428**	0.0314	3/4S
19	REL_03150	F	REL_01804	F	0.32	0.51	0.17	0.212	3/4S	0.0411	**0.0426**	0.0322	3/4S
20	REL_02752	F	REL_04859	F	0.34	0.46	0.20	0.215	3/4S	0.0441	**0.0443**	0.0325	3/4S
21	REL_01502	M	REL_03665	M	0.31	0.48	0.21	0.225	FS	**0.0482**	0.0469	0.0339	FS
22	REL_04592	F	REL_04600	F	0.30	0.48	0.21	0.226	FS	**0.0511**	0.0493	0.0358	FS
23	REL_04693	F	REL_00797	F	0.31	0.47	0.22	0.228	FS	**0.0520**	0.0498	0.0357	FS
24	REL_03607	M	REL_00319	F	0.30	0.49	0.21	0.228	FS	**0.0501**	0.0484	0.0350	FS
25	REL_03220	F	REL_04615	F	0.31	0.46	0.23	0.230	FS	**0.0532**	0.0505	0.0360	FS
26	REL_03212	M	REL_02516	F	0.28	0.53	0.20	0.231	FS	**0.0548**	0.0526	0.0386	FS
27	REL_03310	M	REL_03659	F	0.26	0.56	0.18	0.231	FS	**0.0496**	0.0484	0.0358	FS
28	REL_04427	F	REL_02635	F	0.26	0.54	0.19	0.232	FS	**0.0502**	0.0487	0.0358	FS
29	REL_00122	M	REL_01902	F	0.29	0.49	0.22	0.233	FS	**0.0542**	0.0513	0.0368	FS
30	REL_00284	M	REL_02444	F	0.28	0.51	0.21	0.233	FS	**0.0517**	0.0494	0.0356	FS
31	REL_03838	F	REL_02496	F	0.31	0.45	0.24	0.234	FS	**0.0561**	0.0523	0.0367	FS
32	REL_01564	F	REL_03827	F	0.32	0.43	0.26	0.236	FS	**0.0571**	0.0528	0.0365	FS
33	REL_04529	F	REL_04492	F	0.28	0.50	0.22	0.236	FS	**0.0555**	0.0522	0.0373	FS
34	REL_04494	M	REL_00931	M	0.28	0.49	0.23	0.237	FS	**0.0560**	0.0525	0.0373	FS
35	REL_04466	F	REL_02680	F	0.31	0.43	0.26	0.237	FS	**0.0576**	0.0531	0.0367	FS
36	REL_04405	M	REL_03949	M	0.26	0.52	0.22	0.238	FS	**0.0557**	0.0525	0.0376	FS
37	REL_03880	M	REL_04789	F	0.27	0.50	0.23	0.239	FS	**0.0566**	0.0529	0.0376	FS
38	REL_00383	F	REL_03293	M	0.25	0.53	0.22	0.241	FS	**0.0574**	0.0538	0.0385	FS
39	REL_01888	M	REL_04360	M	0.25	0.54	0.21	0.241	FS	**0.0566**	0.0532	0.0383	FS
40	REL_00792	F	REL_00954	M	0.26	0.51	0.23	0.242	FS	**0.0585**	0.0543	0.0385	FS
41	REL_00872	F	REL_01784	F	0.25	0.53	0.22	0.242	FS	**0.0598**	0.0556	0.0398	FS
42	REL_01450	M	REL_01960	M	0.26	0.51	0.23	0.242	FS	**0.0586**	0.0544	0.0386	FS
43	REL_04616	F	REL_02777	F	0.28	0.47	0.25	0.243	FS	**0.0604**	0.0553	0.0386	FS
44	REL_02899	M	REL_01707	F	0.28	0.45	0.26	0.244	FS	**0.0618**	0.0562	0.0389	FS
45	REL_02905	F	REL_02575	F	0.25	0.52	0.23	0.245	FS	**0.0604**	0.0557	0.0394	FS
46	REL_00769	M	REL_04746	F	0.23	0.57	0.21	0.246	FS	**0.0606**	0.0564	0.0406	FS
47	REL_00009	F	REL_02335	F	0.23	0.55	0.22	0.246	FS	**0.0603**	0.0558	0.0399	FS
48	REL_04475	F	REL_04218	M	0.25	0.51	0.24	0.247	FS	**0.0615**	0.0564	0.0397	FS
49	REL_01150	F	REL_04384	F	0.26	0.49	0.25	0.249	FS	**0.0639**	0.0580	0.0403	FS
50	REL_03944	M	REL_03475	F	0.23	0.54	0.23	0.249	FS	**0.0618**	0.0568	0.0403	FS
51	REL_03904	F	REL_04994	F	0.25	0.50	0.25	0.249	FS	**0.0631**	0.0573	0.0400	FS
52	REL_01654	M	REL_03485	M	0.28	0.43	0.29	0.251	FS	**0.0660**	0.0588	0.0398	FS
53	REL_00504	M	REL_04718	F	0.24	0.50	0.25	0.252	FS	**0.0645**	0.0582	0.0404	FS
54	REL_00339	F	REL_02473	F	0.25	0.48	0.27	0.253	FS	**0.0651**	0.0584	0.0400	FS
55	REL_01016	M	REL_00887	M	0.24	0.50	0.26	0.254	FS	**0.0661**	0.0594	0.0411	FS
56	REL_03977	M	REL_01080	M	0.22	0.54	0.24	0.255	FS	**0.0644**	0.0583	0.0408	FS
57	REL_02339	M	REL_02391	M	0.27	0.44	0.29	0.256	FS	**0.0688**	0.0608	0.0411	FS
58	REL_01524	F	REL_03272	F	0.23	0.51	0.26	0.256	FS	**0.0674**	0.0604	0.0419	FS
59	REL_01285	M	REL_03761	F	0.24	0.50	0.27	0.257	FS	**0.0670**	0.0597	0.0410	FS
60	REL_03395	F	REL_02694	F	0.22	0.52	0.25	0.257	FS	**0.0680**	0.0609	0.0423	FS
61	REL_03151	M	REL_02204	F	0.23	0.50	0.26	0.257	FS	**0.0683**	0.0610	0.0421	FS
62	REL_00968	M	REL_01577	F	0.26	0.45	0.29	0.259	FS	**0.0744**	0.0654	0.0445	FS
63	REL_04439	F	REL_01640	F	0.26	0.43	0.31	0.260	FS	**0.0721**	0.0630	0.0421	FS
64	REL_01546	M	REL_03566	F	0.21	0.53	0.26	0.263	FS	**0.0701**	0.0621	0.0428	FS
65	REL_03442	F	REL_04510	F	0.22	0.51	0.27	0.264	FS	**0.0714**	0.0630	0.0431	FS
66	REL_00340	F	REL_04294	F	0.21	0.53	0.26	0.264	FS	**0.0710**	0.0628	0.0432	FS
67	REL_03001	F	REL_04111	F	0.23	0.48	0.29	0.265	FS	**0.0727**	0.0636	0.0430	FS
68	REL_00282	F	REL_04918	F	0.25	0.44	0.31	0.267	FS	**0.0748**	0.0648	0.0430	FS
69	REL_01083	F	REL_01704	F	0.18	0.57	0.25	0.267	FS	**0.0715**	0.0634	0.0439	FS
70	REL_03388	F	REL_02608	F	0.22	0.50	0.29	0.268	FS	**0.0739**	0.0645	0.0436	FS
71	REL_01924	F	REL_00727	M	0.24	0.45	0.32	0.270	FS	**0.0769**	0.0663	0.0440	FS
72	REL_02208	F	REL_03486	F	0.23	0.46	0.31	0.270	FS	**0.0769**	0.0665	0.0444	FS
73	REL_02718	M	REL_02913	M	0.22	0.48	0.30	0.271	FS	**0.0765**	0.0662	0.0443	FS
74	REL_00634	M	REL_03507	M	0.20	0.51	0.29	0.272	FS	**0.0754**	0.0656	0.0443	FS
75	REL_04741	F	REL_02513	F	0.19	0.52	0.30	0.277	FS	**0.0783**	0.0676	0.0455	FS
76	REL_00601	M	REL_02989	F	0.19	0.51	0.30	0.278	FS	**0.0802**	0.0689	0.0462	FS
77	REL_01624	F	REL_00750	F	0.19	0.51	0.30	0.278	FS	**0.0790**	0.0680	0.0456	FS
78	REL_00824	F	REL_00213	F	0.22	0.45	0.33	0.278	FS	**0.0815**	0.0693	0.0456	FS
79	REL_01264	M	REL_04751	F	0.18	0.52	0.30	0.279	FS	**0.0795**	0.0684	0.0459	FS
80	REL_02208	F	REL_01630	F	0.18	0.52	0.31	0.283	FS	**0.0826**	0.0706	0.0473	FS
81	REL_04704	F	REL_00804	M	0.17	0.52	0.31	0.285	FS	**0.0829**	0.0707	0.0472	FS
82	REL_03627	F	REL_03315	F	0.15	0.55	0.30	0.288	FS	**0.0838**	0.0714	0.0478	FS
83	REL_03486	F	REL_01630	F	0.17	0.50	0.33	0.289	FS	**0.0873**	0.0738	0.0488	FS

FS~UN, 3/4S~UN and 2nd~UN are the LR values for each pair. LR-kinbiplot is the inferred relationship from Graffelman et al. (2019). $\hat{\phi }$: estimated kinship coefficient. ${\hat{k}}_{0}$, ${\hat{k}}_{1}$, and ${\hat{k}}_{2}$: estimated IBD probabilities.

Maximum values of the likelihood ratios of each pair are marked in bold.

## Discussion

In this paper, we show that the likelihood ratio approach is useful for distinguishing three-quarter siblings from FS and 2nd-degree relationships. Figure 4 shows that in a standard $({\hat{k}}_{0},{\hat{k}}_{1})$-plot, 3/4S can easily go unnoticed as FS pairs. The LR approach can be of great help to detect such cases. The LR approach developed in this paper confirmed eight 3/4S pairs previously uncovered by a log-ratio biplot (LR-kinbiplot) approach (Graffelman et al., 2019) for genome-wide SNP array data from the GCAT cohort. The assessment of the precise relationship of a pair based on the numerical values of the LRs, or on a plot of the LRs, ignores the uncertainty in these statistics. We found bootstrap procedures to be extremely useful for quantifying this uncertainty, and consider it to be an invaluable tool for the sensible interpretation of the pairwise LR statistics.

The estimated relationships for the GCAT cohort were to some extent confirmed by an analysis of the surnames of the participants, respecting their privacy. In Spain, people have a double surname, usually the first from the father and the second from the mother. This implies that FS and 3/4S pairs share two surnames, whereas 2nd-degree relationships share only one. All identified 3/4S pairs were confirmed to share two surnames, supporting that these pairs are not 2nd degree.

The proposed LR approach multiplies the likelihoods over loci, under the assumption of independence. The existence of LD between variants violates this assumption. In order to approximately meet the requirement of independence, LD pruning of neighboring variants in a window is therefore recommended (Kling and Tillmar, 2019). This pruning can be done in PLINK (Purcell et al., 2007) or with other software (Calus and Vandenplas, 2018). A future improvement of the LR approach could use Markov chain algorithms (Abecasis and Wigginton, 2005, Kling et al., 2015) that allow efficient likelihood computations on blocks of tightly linked markers.

The LR approach developed in this paper assumes known allele frequencies and non-inbred individuals. The first assumption seems reasonable given the large sample size used in this study. Inbreeding could be accounted for by the use of nine condensed Jacquard coefficients (Hanghoj et al., 2019, Jacquard, 1974) in the development of the likelihood ratio. Inbreeding could yield other levels of relationship in-between FS, 3/4S, and 2nd degree. The $({\hat{k}}_{0},{\hat{k}}_{1})$-plot of the GCAT data in Fig. 4 reveal closeness of the 3/4S and FS pairs, and suggests intermediate relationships like seven-eighths siblings (7/8S) might also exist in the data. Indeed, the full range of 2ND, 5/8S, 3/4S, 7/8S, and FS relationships could be present in the data. It is easily shown that 5/8S and 7/8S have a kinship coefficient of 5/32 and 7/32, respectively. Figure 4 also shows evidence of some pairs in-between a 2nd a 3rd-degree relationship. In future work, the likelihood ratio approach presented in this paper could be further refined to identify all these relationships more precisely. In-between relationships, like the 3/4S relationship studied in this paper, essentially stress that relatedness is a continuous rather than a discrete concept.

### Supplementary information

Supplementary material

## Figures and Tables

**Fig. 8:**
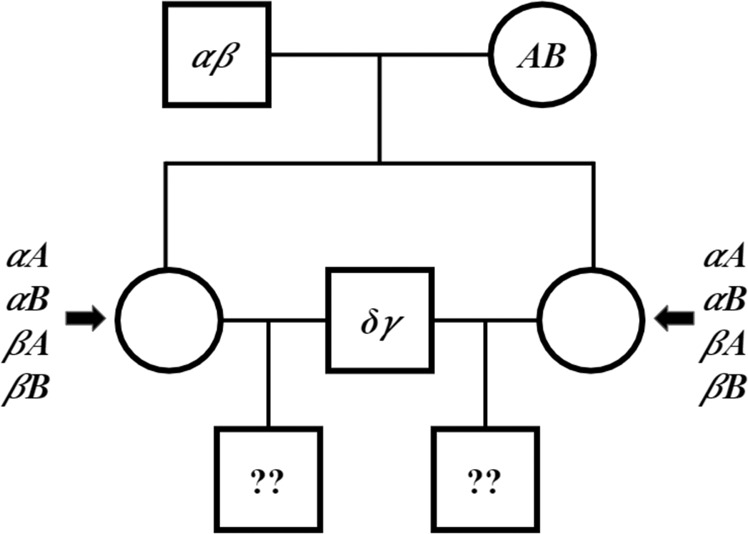
Pedigree of a 3/4S pair where their unshared parents are FS.

## Appendix

### Appendix A

We derive the IBD probabilities for three-quarter siblings (3/4S) in the case that a pair of individuals has one parent in common while their unshared parents are full siblings (FS) (Fig. 8). In the case that the unshared parents have a parent–offspring relationship, the IBD probabilities can be derived analogously.

Let *δ**γ* be the genotype of the common parent of a 3/4S pair, and *α**β*, *α**B*, *A**β*, and *A**B* the possible genotypes of an FS pair. Then, all the possible genotypes and the IBD alleles shared for a 3/4S pair are shown in Table 5.

**Table 5:** Number of IBD alleles for all possible pairs of 3/4S where their unshared parents are FS.

	*α**δ*	*α**γ*	*A**δ*	*A**γ*	*β**δ*	*β**γ*	*B**δ*	*B**γ*
*α**δ*	2	1	1	0	1	0	1	0
*α**γ*	1	2	0	1	0	1	0	1
*A**δ*	1	0	2	1	1	0	1	0
*A**γ*	0	1	1	2	0	1	0	1
*β**δ*	1	0	1	0	2	1	1	0
*β**γ*	0	1	0	1	1	2	0	1
*B**δ*	1	0	1	0	1	0	2	1
*B**γ*	0	1	0	1	0	1	1	2

From Table 5, the IBD probabilities for 3/4S are:

$${k}_{0}=P(IBD=0)=\frac{24}{64}=3/8$$

$${k}_{1}=P(IBD=1)=\frac{32}{64}=1/2$$

$${k}_{2}=P(IBD=2)=\frac{8}{64}=1/8$$

And their kinship coefficient is:

$$\phi ={k}_{1}/4+{k}_{2}/2=\frac{1}{2}\frac{1}{4}+\frac{1}{8}\frac{1}{2}=3/16$$

### Appendix B

Here we show the LR of 3/4S ~ UN for a biallelic SNP whose alleles are *A* and *B*. Let *p* and *q* be the allele frequencies for *A* and *B* of the population under study. For a pair of individuals, we show the LR computation for four genotype pairs: *A**A*/*A**A*, *A**A*/*A**B*, *A**A*/*B**B* and *A**B*/*A**B*. The LR for the remaining genotype pairs (*A**B*/*A**A*, *A**B*/*B**B*, *B**B*/*A**A*, *B**B*/*A**B*, and *B**B*/*B**B*) are equivalent or can be obtained analogously.

The IBD probabilities for 3/4S are (*k*_0_, *k*_1_, *k*_2_) = (3/8, 1/2, 1/8) and for UN pairs are (*k*_0_, *k*_1_, *k*_2_) = (1, 0, 0). Then, according to Tables 1 and 2 and Eqs (1) and (2), the LR for 3/4S ~ UN is derived as follows:

***A******A*****/*****A******A*** **case:**

$$LR=\frac{\frac{3}{8}{p}^{4}+\frac{1}{2}{p}^{3}+\frac{1}{8}{p}^{2}}{{p}^{4}}=\frac{3}{8}+\frac{1}{2p}+\frac{1}{8{p}^{2}}$$

***A******A*****/*****A******B*** **case:**

$$LR=\frac{\frac{3}{8}2{p}^{3}q+\frac{1}{2}{p}^{2}q}{2{p}^{3}q}=\frac{3}{8}+\frac{1}{4p}$$

***A******A*****/*****B******B*** **case:**

$$LR=\frac{\frac{3}{8}{p}^{2}{q}^{2}}{{p}^{2}{q}^{2}}=\frac{3}{8}$$

***A******B*****/*****A******B*** **case:**

$$LR=\frac{\frac{3}{8}4{p}^{2}{q}^{2}+\frac{1}{2}pq+\frac{1}{8}2pq}{4{p}^{2}{q}^{2}}=\frac{3}{8}+\frac{3}{16pq}$$
