# Supplementary material for: A likelihood ratio approach for identifying three-quarter siblings in genetic databases
Source: Heredity (Edinb). 2021 Jan 15;126(3):537–47. doi: 10.1038/s41437-020-00392-8 (PMC8027836; doi:10.1038/s41437-020-00392-8)
Supplement: Supplementary file 1 — Supplementary material [file 41437_2020_392_MOESM1_ESM.pdf]

Supplementary Figure

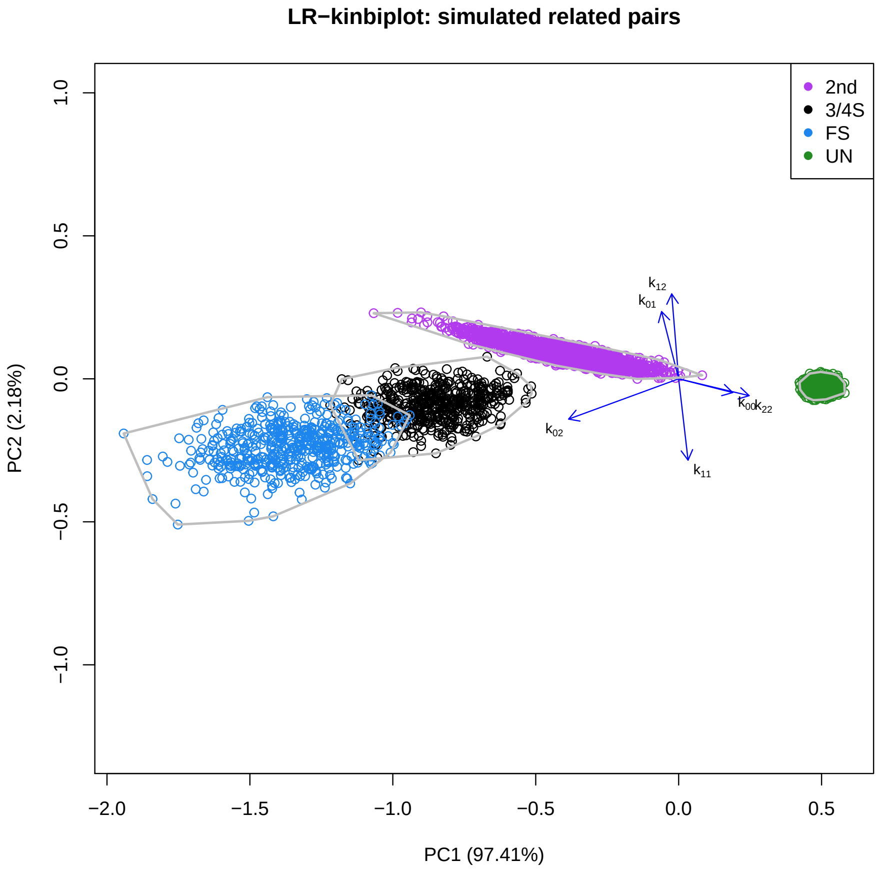

Figure S1: Log-ratio biplot with convex hulls for relationships of simulated pairs.
